# Impact of the Extraction Method on the Chemical Composition and Antioxidant Potency of *Rosmarinus officinalis* L. Extracts

**DOI:** 10.3390/metabo13020290

**Published:** 2023-02-16

**Authors:** Nedra Dhouibi, Simona Manuguerra, Rosaria Arena, Concetta Maria Messina, Andrea Santulli, Seifeddine Kacem, Hatem Dhaouadi, Abdelkarim Mahdhi

**Affiliations:** 1 Département de Chimie, Faculté des Sciences, Université de Monastir, LR21ES04-Chimie de l’Environnement & des Procédés Propres, Bvd de l’Environnement, Monastir 5000, Tunisia; 2 Laboratorio di Biochimica Marina ed Ecotossicologia, Dipartimento di Scienze della Terra e del Mare DiSTeM, Università degli Studi di Palermo, Via G. Barlotta 4, 91100 Trapani, Italy; 3 Istituto di Biologia Marina, Consorzio Universitario della Provincia di Trapani, Via G. Barlotta 4, 91100 Trapani, Italy; 4 AgriLand, Avenue Habib Bourguiba, Tunis 2000, Tunisia; 5 Laboratory of Analysis, Treatment and Valorization of Pollutants of the Environment and Products, Faculty of Pharmacy, University of Monastir, Monastir 5000, Tunisia

**Keywords:** *Rosmarinus officinalis* L., antioxidant, UPLC-MS-DAD analysis, sonication, supercritical extraction

## Abstract

**Highlights:**

- Ultrasonic extract of rosemary with ethanol was very rich in polyphenols
- Higher antioxidant ability was observed at short time extraction (10 mins)
- Protective effect against oxidative stress was tested in HS-68 fibroblast cell lines
- Strong protective effect was registered at low concentrations.

**Abstract:**

*Rosmarinus officinalis* L. is a dietary source that produces polyphenols as secondary metabolites. These natural compounds with potent antioxidant abilities are increasingly recommended as a supplement to inhibit oxidative stress. In the current work, we evaluated the impact of the extraction method on the chemical composition of *R. officinalis* extract, especially on the content of carnosic (CA) and rosmarinic (RA) acids using UPLC-MS-DAD as well as on their antioxidant potency. Four extracts of Tunisian rosemary were obtained from non-conventional extraction techniques:ultrasound-assisted extraction (UAE),supercritical extraction (SFE) and UAE and SFE combined ((UAE-SFE(I), UAE-SFE(II)). The UAE exhibited the best total phenolic compounds (i.e., 85.27 mg GAEg^−1^), the highest content of CAand RA and the strongest antioxidant abilities (i.e., IC_50_ = 0.13 mg/mL and EC_50_ = 0.93 mg/mL for DPPH scavenging test and iron reducing power ability assay). The evaluation of antioxidant activity of UAE inhuman skin fibroblast (HS-68) cell line was carried out after the induction of oxidative stress. The results determined by 3-(4,5-dimethylthiazol-2-yl)-2,5-diphenyltetrazolium bromide (MTT) assay showed a strong protective effect against H_2_O_2_oxidative stress induction in cells pretreated with UAE. The obtained results allow us to give new insight about the effect of the extraction method on the chemical composition and biological activities of the extract and the importance of the choice of the most appropriate processing technique to prepare rosemary extract with a high antioxidant potency and protective effect against oxidative stress.

## 1. Introduction

Rosemary (*Rosmarinus officinalis* L.) is a xeromorphic plant that belongs to the family of *Lamiaceae* [1]. It grows spontaneously on mountains, cliffs and stony places, near the sea, particularly in the Mediterranean basin, and is widespread in Africa, Europe and Asia [2]. *R. officinalis* L. is an aromatic herb that exhibits substantial antioxidant activity via its free radicals, reactive oxygen species (ROS) and its scavenging capability. This is because of the high level of polyphenolic compounds that act as natural potent antioxidants to inhibit or delay lipid oxidation in food products [3]. Among polyphenols, the predominant ones are phenolic diterpenes (carnosol, rosmanol, rosmadiol, methylcarnosate), phenolic acids (carnosic acid, rosmarinic acid and hydroxycinnamic acid ester) and flavonoids [4,5,6]. Rosemary can be used dried, fresh, as an essential oil or as an extract. It is often used in culinary applications as a natural ingredient to adjust the flavor in tea infusions and cooking [7] and in folk medicine as diuretic [8], analgesic [9], anti-inflammatory [10] and antidepressant [11]. In addition to being recognized by its antioxidant power, rosemary extracts exhibited numerous biological activities, such as anti-cancer [12], anti-diabetogenic [13], antinociceptive [14], antifungal [15] and antibacterial [16] effects. Among the antioxidant compounds identified in rosemary extracts, we found carnosic acid (CA) and rosmarinic acid (RA), known to possess the most potent antioxidant properties, as well as their derivatives carnosol, rosmanol and galdosol [3]. CA is an abietane phenolic diterpene belonging to the terpene classification (also termed terpenoid or isoterpene) which is one of the largest class of plant secondary metabolites. Because of its O-phenolic hydroxyl groups, CA is classified among polyphenols [17]. RA is an ester of (R)-(+)-3-(3,4-dihydroxyphenyl) lactic acid and caffeic acid and belongs to the family of hydroxycinnamic acids [18]. Great attention has been paid to natural dietary products, particularly those of the rosemary plant, as they are considered potent antioxidants. However, despite the great virtues that these molecules possess, their extraction and conservation are relatively delicate since they are thermolabile, photosensitive and prone to chemical changes. The oxidation or/and degradation of the bioactive compounds in rosemary extract could be avoided using non-thermal treatment technology methods to produce rosemary extracts (e.g., supercritical fluid extraction and sonication). These techniques operating at low temperatures and short extraction times are highly suitable for the extraction of thermo/photolabile molecules such as antioxidants [19]. Supercritical fluid extraction (SFE) is an innovative emerging approach to obtain bioactive substances which avoids some of the drawbacks of traditional solvent extraction techniques and is considered to beenvironmental-friendly technology [20]. Particularly, CO_2_ is the most commonly used solvent in SFE for being non-toxic and inert, with lower supercritical temperature and pressure, solvent properties and easy removal [21]. In previous years, the SFE process was increasingly applied in the food industry as the ideal tool in the extraction of aromatic plants [22]. In addition to SFE, ultrasound-assisted extraction (UAE), also termed sonication, is one of non-thermal treatment technologies that has been extensively used for extracting bioactive compounds from medicinal plants [23]. This treatment has been reported to be simple, inexpensive, reliable, along with observably enhancing the extract yield and quality and inhibiting bioactive substances damage [24]. Furthermore, sonication is considered advantageous because of its short processing time with reduced energy consumption. For the abovementioned benefits, this technique is considered an environment-friendly and effective alternative to conventional extraction methods [25]. To overcome the negative repercussions of the conventional methods of extraction of bioactive substances and taking into consideration the concept of “green chemistry”, we have chosen in the current work to study Tunisian rosemary extracts obtained with ultrasound and supercritical extractions. The impact of the extraction method on the chemical composition of *R. officinalis* extract, and especially on the content of CA and RA using UPLC-MS-DAD, as well as on their antioxidant potency, was evaluated based on a DPPH radical test, iron reducing power and their protective effect on human fibroblast cells against oxidative stress.

## 2. Materials and Methods

### 2.1. Plant Material, Reagents and Chemicals

*R. officinalis* L. was collected during the flowering season from its natural habitat Sbikha Village/Maarouf, Kairouan (Tunisia). The aerial part of rosemary was dried at 50–60 °C in shade until moisture content reached 8.4%. Then, the dried plant material was finely ground using a laboratory mill. The obtained particle sizes were in the range of 500 to 1000 µm. Absolute ethanol (≥99.8%), methanol (99.8%), acetic acid (≥99.7%), Carnosic acid (≥95.0%) and Rosmarinic acid (≥98%) were bought from Sigma-Aldrich (Monastir, Tunisia). Gallic acid, DPPH, Folin–Ciocalteu reagent, iron(III) chloride (FeCl_3_), human skin fibroblasts (HS-68) and 3-(4,5-dimethylthiazol-2-yl)-2,5-diphenyltetrazolium bromide (MTT), Dulbecco’s Modified Eagle’s Medium (DMEM), glutamine, fetal bovine serum and penicillin–streptomycin were purchased from Merck KGaA (Darmstadt, Germany).

### 2.2. Preparation of the Extracts

#### 2.2.1. Ultrasound Assisted Extraction

The sonication was performed in an Ultrasound probe using a sonotrode (UIP1000hd from Hielscher technologies, Teltow, Germany) [25]. A mixture of powdered rosemary and absolute ethanol (1:3 *w*/*v*) was placed in a double jacket reactor and exposed to ultrasound at 750 W and 5 kHz for 10 min. The extraction temperature was regulated at 25 °C. The ultrasonic extract was centrifuged at 4000 rpm for 10 min. The supernatant was removed and concentrated under a vacuum by using a rotary vacuum evaporator (HeivapPresición HL G3 from Heidolph technologies). The dried extract (UAE) was then weighed and stored at +4 °C in an amber vial until further analysis.

#### 2.2.2. Supercritical Fluid Extraction

The supercritical extraction was carried out using carbon dioxide in a pilotplantscale supercritical fluid extractor (Aerospace Technology, Zunyi, China, model SUS304) comprising two-cylinder extraction cells (1 L and 5 L) and two separators (S1 and S2). The extractor unit (50 mm inner diameter and 250 mm length) was charged with 100 g of dried ground rosemary. The extraction was performed at P = 15 MPa, T = 45 °C, 7% (*w*/*w*) ethanol for 180 min and the CO_2_ flow rate maintained constant (20 L·h^−1^) [25], with slight modifications. The supercritical extract (SFE) was weighed and stored in darkness at +4 °C.

#### 2.2.3. Re-Extraction of UAE with Supercritical CO_2_

Another extraction technique was tested in this study combining the two previous extraction methods. The ground rosemary was first sonicated with ethanol, and then the residue was exposed to supercritical extraction under two different supercritical extraction conditions (i.e., pressure, % co-solvent and CO_2_ flow rate). The first extraction (UAE-SFE(I)) was performed under the following working conditions (7% co-solvent, P = 15 MPa, flow-rate = 20 L/h), the second extraction (UAE-SFE(II)) was conducted using neat CO_2_ (0% co-solvent, P = 15 MPa, flowrate = 20 L/h). The two extractions were conducted at 45 °C during 180 min. The obtained extracts were collected and refrigerated at +4 °C prior to handling.

### 2.3. Total Phenolic Content (TPC)

The evaluation of TPC in rosemary extracts was accomplished according to Folin and Ciocalteu reported by Dhouibi et al. [25] using gallic acid (GAE) as standard. An aliquot of 25 μL of each extract reconstituted in ethanol (4000 µg·mL^−1^) were added to a volume of 12.5 µL Folin–Ciocalteu reagent diluted in ethanol (1:1) and 150 μL of milliQ water. The mixtures were vigorously shaken at room temperature in darkness for 5 min. Then, 25 μL of Na_2_CO_3_ (2% *w*/*v*) solution was added and the mixtures were again incubated at room temperature in darkness for 1 h by intermittent shaking. The absorbance was measured using UV/Vis spectrophotometer (Varian Cary 50 Scan, Palo Alto, CA, USA) at 725 nm. TPC values (average of triplicate) were presented as gallic acid equivalents (mg _GAE_ g^−1^).

### 2.4. DPPH Scavenging Activity

The power of rosemary extracts to neutralize free radicals was evaluated using DPPH assay as described byMessina et al. [26]. Briefly, an aliquot of 40 μL of ethanol mixture containing 0.125 to 4 mg·mL^−1^ of rosemary extract was added to 160 μL of ethanol solution of DPPH (0.1 mM) prepared daily, shaken vigorously at room temperature and then incubated for 30 min in darkness. Absorbance was recorded at 515 nm. The DPPHscavenging activity of each sample was then calculated as percent inhibition based on the following equation:

% inhibition = 100 (A_blank_ − A_sample_)/A_blank_ (1)

Antioxidant power of tested extract was expressed as the mean of IC_50_ ± SD, where IC_50_ is defined as the Inhibitory Concentration that causes a decrease to half of the absorbance.

### 2.5. Iron Reducing Power Activity

The reducing power of rosemary extracts was evaluated based on spectrophotometric detection of iron reduction method as reported by Manuguerra et al. [27]. Aliquots (300 µL) of rosemary ethanolic solutions at different concentrations were mixed with an equal amount (300 µL) of phosphate buffer (0.2 M, pH 6.6) and potassium ferricyanide [K_3_Fe(CN)_6_] (1% *w*/*v*); the mixture was incubated at 50 °C for 20 min. Then, 300 µL of trichloroacetic acid (1% *w*/*v*) was added and the mixture was centrifuged for 10 min at 3000 rpm. Distilled water (50 µL) and iron trichloride (100 µL, 0.1% *w*/*v*) were added to 50 µL of the upper layer of the rosemary solution. Absorbance was recorded at 700 nm. The effective concentration corresponding to the absorbance = 0.5 (EC_50_) was determined from linear regression analysis.

### 2.6. Quantitative Analysis of Carnosic and Rosmarinic Acidsby UPLC-MS-DAD

Samples were reconstituted in methanol with a concentration of 10 mg/mL, filtered through a 0.45 μm cellulose filter and submitted to UPLC-MS-DAD analysis. Chromatographic analyses were accomplished to quantify CA and RA content in the rosemary extracts by the means of ultra-performed liquid chromatography Waters ACQUITY QSM (Milford, CT, USA) equipped with a Waters Acquity PDA photodiode array detector (UPLC eLambda 800 nm), coupled with mass spectrometry equipped with a single Quadrupole Detector (SQD2). Analyses were performed using a C18 column (XBridgeTM BEH, 2.5 µm 2.1 × 50 mm) (Waters, Milford, CT, USA). The mobile phase consisted of 40% water + 60% acetonitrile + 0.1% Formic acid (solvent A) and water (solvent B) applying the following gradient: from 0 to 2 min, 30% A; increasing from 2 to 6 min up to 100% A, from 6 to 10 min, 100% B and from 10 to 12 min, initial conditions were reached at 100% A. The UV detection was carried out at 230 and 328 nm as CA and RA wavelengths, respectively. Calibration curves were prepared with pure standards at concentrations of 1 to 100.0 μg/mL for rosmarinic acid and 1 to 400.0 μg/mL for carnosic acid. Linearity was assessed by calculating the correlation coefficient R^2^ of the calibration curve determined from a range of concentrations of each standard. Selectivity was assessed by examining each chromatogram to confirm that no compound could interfere with the analyte. The results were expressed in mg CA/g extract and in mg RA/g extract.

### 2.7. Cell Culture

Human skin fibroblast cells HS-68 (ECACC n◦ 89051701, Sigma-Aldrich, Saint Louis, MO, USA) were cultured in in Dulbecco’s Modified Eagle’s Medium (DMEM) supplemented with 2 mM glutamine, 10% (*v*/*v*) inactivated fetal bovine serum (FBS) and 100 μg/mL penicillin–streptomycin and incubated at 37 °C in 5% CO_2_ humidified atmosphere under sterile conditions using a grade (II) flow hood.

#### Cytotoxic and Protective Effect against H_2_O_2_-Induced Oxidative Stress in HS-68 Cells

A preliminary test on fibroblast cells was carried out to evaluate the effects of different concentrations of UAE on cell viability and to individuate the adequate range of concentrations to perform the experiment of H_2_O_2_-oxidative stress induction. UAE (dissolved in ethanol) was diluted in culture medium at a concentration rangeof 0.02–0.32 µg/mL, at a final solvent concentration not exceeding 0.1% (*v*/*v*). Briefly, confluent cells were trypsinized and seeded in a 96-well plate at a density of 7 × 10^3^ cells/well and incubated for 24 h, before the exposure to UAE. After 24 h, cells were treated with different concentrations of UAE (0.02–0.32 µg/mL) and after 24 h, cell viability was evaluated.

The UAE concentration that does not induce cell mortality (0.16 µg/mL) was selected for the next trial. After 24 h of treatment, the cells were exposed to 50 µM of hydrogen peroxide and incubated for 2 h at 37 °C. Additional wells were treated with natural gallic acid (GAE) and synthetic antioxidant N-acetilcysteine (NAC 10 µM) with the same concentration, and another set of cells was treated only with the promoter of oxidative stress (HP). The viability was evaluated using 3-[4,5-dimethylthiazol-2-yl]-2,5-diphenyltetrazolium bromide (MTT) assay as described by Messina et al. [26]. Tests were performed in quintuplicate. The results are expressed as percentage of viable cells with respect to controls.

### 2.8. Statistical Analysis

All experiments were performed in triplicate and the results are presented as data average ± SD. The one-way analysis of variance (ANOVA) was used to investigate statistical evaluation for each sample followed by the Student–Newman–Keuls test. The homogeneity of variance was confirmed by the Levene test and values with (*p* < 0.05) were considered significant. All measurements were analyzed by SPSS^®^ software (version 20, SPSS Inc., Chicago, IL, USA).

## 3. Results and Discussion

### 3.1. Total Phenolic Content (TPC)

**Figure 1:**
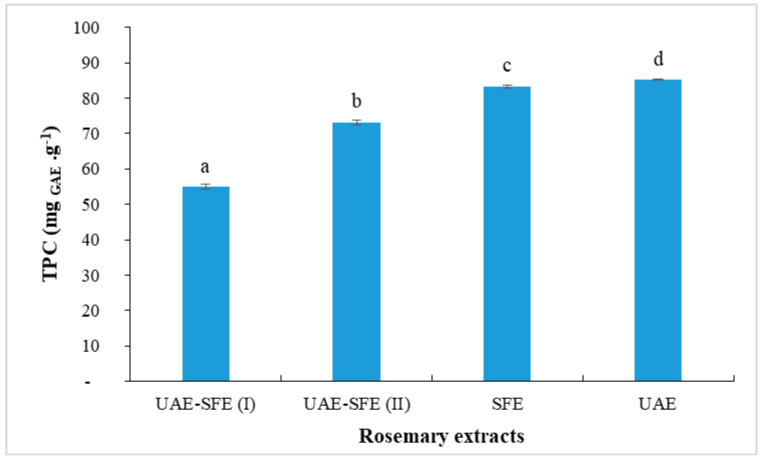
Total phenolic content (mg _GAE_/g) in rosemary extracts (Different letters indicate significant difference (*p* < 0.05)).

Rosemary extracts contained phenolic compounds that exert antioxidant activity through their abilities to scavenge free radicals and therefore prevent lipid oxidation reaction [28]. Thus, the assessment of TPC is primordial. The results of TPC in rosemary extracts are shown in Figure 1. The obtained values ranged from 55 to 85 mg _GAE_·g^−1^. As far as our literature review could ascertain, our UAE of *R. officinalis* extracts exhibited a total phenolic content higher than those reported in the literature previously. The ultrasonic extract obtained with ethanol showed aTPC = 49.14 mg _GAE_·g^−1^ [29]. Zeroua et al. [30] reported that the ethanolic extract of *R. officinalis* had 17.32 and 13.31 mg _GAE_·g^−1^, for the extract obtained with Soxhlet extraction and maceration, respectively. Sharma et al. [31] studied different extracts of *R. officinalis* obtained by decoction and Soxhlet extraction, including fermentation and sonication; the best TPC (76.64 mg _GAE_ g^−1^) content was detected in Soxhlet extract obtained with methanol.

As shown in Figure 1, the highesttotal phenolic concentration was recorded when ethanol was used as a solvent in UAE (85 mg _GAE_ g^−1^) and as co-solvent in SFE (83 mg _GAE_ g^−1^). These results are justified by the affinity between polyphenols, as polar compounds, with a polar solvent (i.e., ethanol). Omar et al. [20] have reported that the extraction capacity of polar molecules such as polyphenols can be improved when co-solvent was used. However, even the extract UAE-SFE(I) was obtained with ethanol as co-solvent, it presented the lowest TPC = 55 mg _GAE_ g^−1^. This finding could be explained by the fact that the rosemary plant was previously extracted by ultrasonic extraction with ethanol and thus, polyphenols were almost fully extracted in the first step. Additionally, the residual polyphenols could hardly be extracted at the tested pressure (15 MPa) and temperature (45 °C). Under these conditions, the extraction of volatile compounds is rather favored. It is known that sonication with ethanol presents the most effective method for the extraction of polyphenols; nevertheless, under the conditions tested, the residual rosemary is not completely exhausted by sonication, a considerable content of phenolic compounds still remains in the residue after ultrasonic extraction, which explains the considerably high content obtained in UAE-SFE(II) (TPC = 73 mg _GAE_ g^−1^). Overall, the sonication observably promotes the extraction of antioxidants with respect to supercritical extraction. For that, an optimization of the sonication process is necessary to obtain a polyphenol-rich rosemary UAE.

### 3.2. DPPH Scavenging Activity

DPPH^•^ is a stable radical, which has been commonly considered as a tool to assess the free radical scavenging power of an antioxidant [1]. This radical is readily reduced by an antioxidant (AH) as demonstrated by the following reaction [32].

DPPH^•^ + AH → DPPH − H + A^•^ (2)

**Figure 2:**
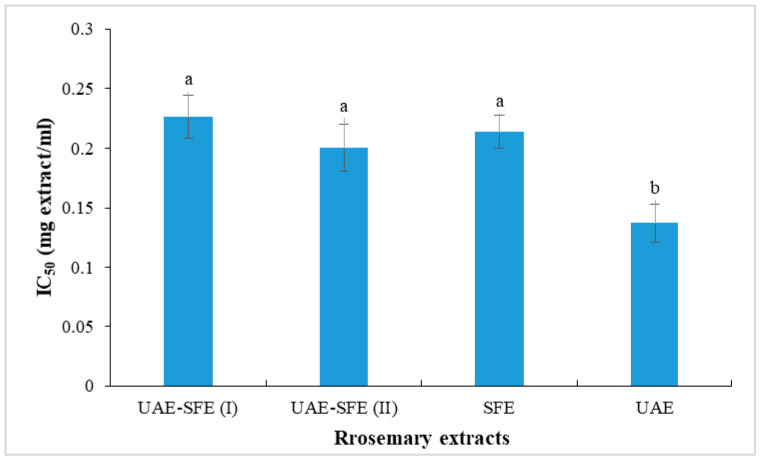
DPPH radical scavenging activity (IC_50_, mg/mL) of rosemary extracts. Different letters indicate significant difference (*p* < 0.05).

The disappearance of the DPPH radical was spectrophotometrically measured at 515 nm and considered as an assessment of antioxidant activity. In the current paper, the antioxidant ability of test extracts was evaluated based on their IC_50_ values determined from a calibration curve obtained by plotting % inhibition as a function of concentration of the test sample. IC_50_ values of rosemary extract are presented in Figure 2. All the studied rosemary extracts (UAE, SFE, UAE-SFE(I) and UAE-SFE(II)) were capable of neutralizing DPPH^•^ radicals, with similar capacities recorded in UAE-SFE(I), UAE-SFE(II) and SFE samples (IC_50_ = 0.22, 0.20, 0.21 mg/mL, respectively). The lower the IC_50_, the higher the antiradical ability. Interestingly, UAE exhibited the highest DPPH radical scavenging ability with the lowest IC_50_ = 0.13 mg/mL. This result indicated the important effect of solvent and the extraction methods. The noticeable decrease in DPPH scavenging ability could be explained by the lack of hydrosoluble phenolic compounds in SFE extracts [22].

### 3.3. Reducing Power Activity

**Figure 3:**
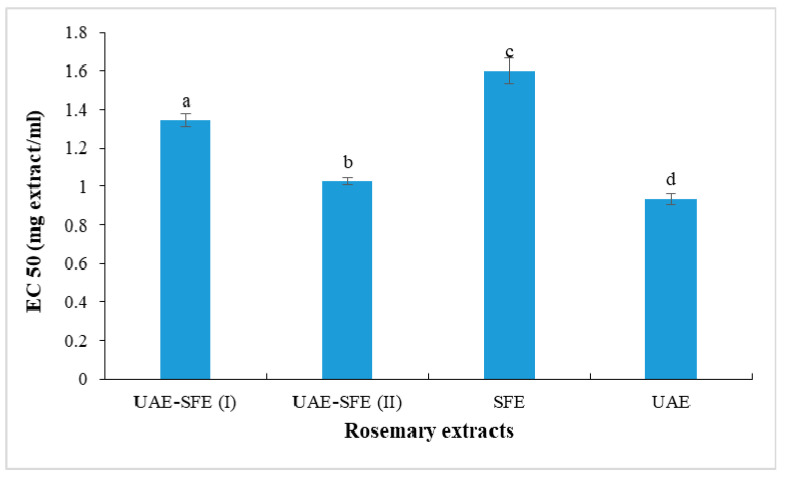
Reducing power (EC_50_, mg/mL) of rosemary extracts. Different letters indicate significant difference (*p* < 0.05).

The ferric reducing power was evaluated spectrophotometrically at 700 nm based on the measurement of iron reduced form concentration in the presence of rosemary extract. The obtained results in terms of EC_50_ (defined above as the effective concentration to reduce 50% of the absorbance) were presented in Figure 3. The stronger ability was registered in UAE with the lowest EC_50_ = 0.93 ± 0.03 mg/mL. The changes in ferric reducing properties values followed asimilar trend as discussed above in DPPH scavenging assay: SFE extract < UAE-SFE extract < UAE extract. These findings could be explained by the fact that the SFE under the working conditions rather favored the extraction of lipophilic constituents endowed with lower antioxidant effects compared with those of polar compounds that are extracted with ethanol in UAE.

### 3.4. Protective Effect on Human Fibroblast Cells (HS-68)

Despite the important antioxidant power of rosemary UAE, its potential activity in fibroblast human cells has not been reported yet. In this respect, this study examinedthe protective effect of the selected rosemary extract (UAE) in HS-68 cell line exposed to H_2_O_2_ oxidative stress.

**Figure 4:**
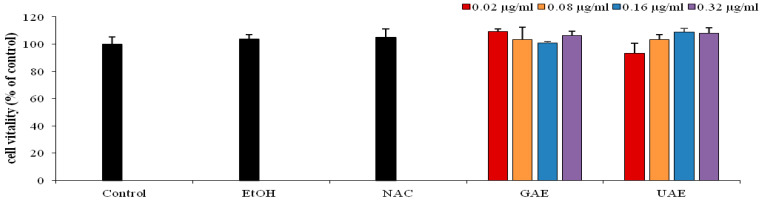
Percentage of viability of HS-68 fibroblast cells, obtained by MTT test (all data are presented as mean ± SD; *n* = 5). Control (cells without treatment), cells treated with different concentrations of rosemary UAE (0.02, 0.08, 0.16 and 0.32 µg/mL) (0.1% < ethanol) for 24 h.

Cell proliferation was evaluated by MTT assay (*p* < 0.05) after pretreatment with different concentration of UAE (0.02–0.32 µg/mL) for 24 h. Thepreliminary results showed that the increased concentration of UAE did not induce cytotoxic effect in HS-68 cells (Figure 4).

In the next trial, we chose the concentration of 0.16 µg/mL UAE to evaluate the protective effect against a chemical promoter of oxidative stress, hydrogen peroxide.

**Figure 5:**
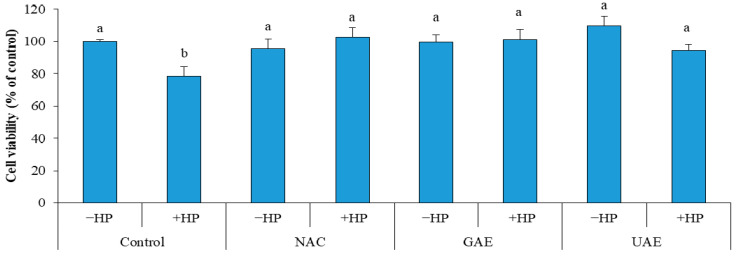
Effect of rosemary UAE on HS-68 fibroblast cells exposed to oxidative stress, induced by H_2_O_2_.HP = hydrogen peroxide; NAC = cells pretreated with the synthetic antioxidant N-acetyl cysteine; GAE= cells pretreated with gallic acid; UAE = cells pretreated with ultrasonic extract of rosemary. Different letters indicate significant difference (*p* < 0.05).

After treatment of cells with hydrogen peroxide, a considerable decrease (11.4%) in cell viability of control was recorded (Control + HP). Whereas, no decrease in cell viability was observed in cells pretreated with UAE (UAE + HP), which indicate a strong protective effect against oxidative stress, similar to that of GAE (GAE + HP) and NAC (NAC + HP) as illustrated in Figure 5. The results demonstrated the prominent bioactive effects (i.e., inhibition of lipid oxidation and prevention of ROS-induced damage). Consequently, the rosemary UAE could be used in culinary, pharmaceutical and cosmetics applications [33].

### 3.5. Correlation between Carnosic and Rosmarinic Acids Content and Antioxidant Potency

With the purpose to quantify the major antioxidants present in rosemary, the extracts were analyzed by UPLC-MS-DAD. Contents of CA and RA are expressed as a mean of mg/g _extract_ and presented in Table 1. In general, results showed that CA was more abundant than RA. In fact, CA concentration ranged from 10.5 mg/g extract for UAE-SFE(II) to 62.19 mg/g extract for UAE, while RA content varied from 0.0 for SFE and 54.14 mg/g extract for UAE in the dried extract. A lower content of CA was registered only in the extract obtained without polar solvent. This finding was expected since the CA is considered a polar antioxidant with two phenolic hydroxyl groups. CA was extracted in SFE at the rate of 16.33 mg/g _extract_. According to Omar et al. [20], even when working under middle CO_2_ density (pressure 15 MPa), oxygenated terpenes, in this case CA, are soluble in supercritical CO_2_. However, the unexpected result was that RA, which is more polar with four phenolic hydroxyl groups, was absent in SFE extraction carried out with 7% of ethanol as co-solvent (SFE). This result could be explained by the decrease of selectivity when a higher amount of a polar co-solvent was added [32]. Moreover, the surprising amounts of CA and RA obtained in SFE could be attributed to the matrix effects which make a quantitative extraction of target molecules by SFE difficult. Significantly, the highest concentrations of CA and RA were recorded in UAE. With regard to antioxidant potency, based on the DPPH and iron reducing power assays, the best results were registered in UAE presenting the highest TPC, particularly CA and RA. Furthermore, the significant protective effect against oxidative stress similar to GA and NAC could be mainly due to the high content of CA and RA, known by their important antioxidant activity in cells as previously reported by [3]. The recorded antioxidative response of UAE is probably based on the contribution of CA highly abundant in rosemary as compared to other phenolic diterpenes. As reported by Birtić et al. [17], the free antiradical activity of CA is attributed to the two O-phenolic hydroxyl groups at C11 and C12 and follows a mechanism similar to that of α-tocopherol. Additionally, low amounts of these compounds were recorded in UAE-SFE(I) and UAE-SFE(II) which could be explained by the fact that, in both extractions, the rosemary was previously extracted with sonication and hence, the targeted antioxidants were almost exhausted. Remarkably, although the two extracts UAE-SFE (I) and SFE were obtained with an equal dose of ethanol, the contents of CA and RA were all the more important when the plant matrix undergoes a sonication before the supercritical extraction. In this sense, the sonication could be considered as a pretreatment of the rosemary plant material to further reduce the particle size by milling and breaking cells [34]. The enhancement of antioxidant extraction could be attributed to the following: firstly, ultrasound burst cell walls causing collapsed cavitation bubbles which improves inner mass transfer; secondly, cavitational collapse allows better contact between the plant material and the extraction solvent; and finally, the bursting of the cells produced an ultrasonic jet solvent which forces it to access inside cells and dissolve the components [35].

## 4. Conclusions

This study was performed to compare the effect of supercritical and ultrasound extraction processes on chemical composition and antioxidant potency of extracts from Tunisian *R. officinalis* L. Sonication presents great potential as a green method for obtaining antioxidant-rich rosemary extract with higher concentrations of polyphenolic constituents. Findings demonstrated that sonication is more effective in the extraction of carnosic and rosmarinic acids. It was revealed that, among the studied extracts, rosemary UAE exhibited higher antioxidant activity according to both antioxidant activity assays. This was related to the higher TPC, and particularly, CA and RA contents. Moreover, tested in fibroblast human cells exposed to oxidative stress, our UAE performed a strong antioxidant activity compared to GAE and NAC. Hence, aiming to substitute synthetic antioxidants, our rosemary UAE, a natural source of antioxidants, may be of interest for several industrial applications.

The obtained results of the current project will be applied on an industrial scale and future research will focus on the optimization of sonication in order to obtain extracts rich in antioxidants and ultimately isolate CA and RA. However, biological tests are required to study the application of UAE in nutritional, pharmaceutical and cosmetics formulation.

## Figures and Tables

**Table 1:** CA and RA content in *R. officinalis* L. extracts.

Samples	Extraction Conditions	Carnosic Acid (mg/g _extract_)	Rosmarinic Acid (mg/g _extract_)
UAE-SFE(I)	750 W and 5 kHz, 10 min, 25 °C, material/solvent ratio 1/10 + P = 15 MPa, t = 180 min, CO_2_ flow-rate = 20 L/h, 7% (*w*/*w*) co-solvent.	22..93 ± 0.739	1.48 ± 0.047
UAE-SFE(II)	750 W and 5 kHz, 10 min, 25 °C, material/solvent ratio 1/10 + P = 30 MPa, t = 180 min, CO_2_ flow-rate = 18 L/h No co-solvent	10.50 ± 0.462	1.60 ± 0.070
SFE	P = 15 MPa, t = 180 min, CO_2_ flow-rate = 20 L/h, 7% (*w*/*w*) co-solvent.	16.33 ± 0.129	00.0
UAE	750 W and 5 kHz, 10 min, 25 °C, material/solvent ratio 1/10	62.19 ± 0.903	54.14 ± 0.786

Data are expressed as mean ± S.D (*n* = 3). one-way ANOVA *p* < 0.001 followed by a homogeneity Levene test: *p* < 0.05 compared with gallic acid.

## Data Availability

Data sharing not applicable: No new data were created or analyzed in this study. Data sharing is not applicable to this article.
